# Construction of a bacteriophage-derived recombinase system in *Bacillus licheniformis* for gene deletion

**DOI:** 10.1186/s13568-023-01589-w

**Published:** 2023-08-26

**Authors:** Fang Xue, Xufan Ma, Cheng Luo, Dongliang Li, Guiyang Shi, Youran Li

**Affiliations:** 1 Key Laboratory of Chinese Cigar Fermentation, Cigar Technology Innovation Center of China Tobacco, Tobacco Sichuan Industrial Co., Ltd, Chengdu, 610000 P. R. China; 2 National Engineering Research Center for Cereal Fermentation and Food Biomanufacturing, Jiangnan University, 1800 Lihu Avenue, Wuxi, Jiangsu 214122 P. R. China; 3 Jiangsu Provincial Engineering Research Center for Bioactive Product Processing, Jiangnan University, 1800 Lihu Avenue, Wuxi, Jiangsu 214122 P. R. China

**Keywords:** *Bacillus licheniformis*, Homologous recombination, Recombinase RecT

## Abstract

*Bacillus licheniformis* and its related strains have found extensive applications in diverse industries, agriculture, and medicine. However, the current breeding methods for this strain primarily rely on natural screening and traditional mutagenesis. The limited availability of efficient genetic engineering tools, particularly recombination techniques, has hindered further advancements in its applications. In this study, we conducted a comprehensive investigation to identify and characterize a recombinase, RecT, derived from a *Bacillus* phage. Remarkably, the recombinase exhibited a 10^5^-fold enhancement in the recombination efficiency of the strain. To facilitate genome editing, we developed a system based on the conditional expression of RecT using a rhamnose-inducible promoter (P_rha_). The efficacy of this system was evaluated by deleting the *amyL* gene, which encodes an α-amylase. Our findings revealed that the induction time and concentration of rhamnose, along with the generation time of the strain, significantly influenced the editing efficiency. Optimal conditions for genome editing were determined as follows: the wild-type strain was initially transformed with the genome editing plasmid, followed by cultivation and induction with 1.5% rhamnose for 8 h. Subsequently, the strain was further cultured for an additional 24 h, equivalent to approximately three generations. Consequently, the recombination efficiency reached an impressive 16.67%. This study represents a significant advancement in enhancing the recombination efficiency of *B. licheniformis* through the utilization of a RecT-based recombination system. Moreover, it provides a highly effective genome editing tool for genetic engineering applications in this strain.

**Supplementary Information:**

The online version contains supplementary material available at 10.1186/s13568-023-01589-w.

## Introduction

*Bacillus licheniformis* is a gram-positive bacterium that has high application value due to its simple fermentation conditions, comprehensive enzyme systems, high enzyme production, and food safe characteristics (Li et al. 2018b). Currently, *B. licheniformis* has been widely used in industry for the production of peptide antibiotics (such as bacitracin and proteins), organic acids, and polymers (such as citric acid, guanosine monophosphate, and polyglutamic acid) (He et al. 2023; Xiao et al. 2023), it also has important applications in aquaculture, agriculture, biomedical and pharmaceutical fields. However, the progress in studying *B. licheniformis* through genetic modification or expanding its industrial applications is still very limited. The main reason for this is the extremely low transformation and homologous recombination (HR) efficiency of this strain, compared to that of other bacterial species such as *Escherichia coli* and *Bacillus subtilis* (Li et al. 2020). Therefore, there is an urgent need to develop efficient genetic engineering tools.

With the rapid development of biotechnology, it is no longer simply possible to obtain genetic information from organisms, but it is now possible to envision directed modifications to the genome sequence of organisms through genome editing technology, enabling them to possess the desired phenotype or express specific products. Genome editing technology is used to design specific modifications in target gene sequences, including insertion, deletion, loss, replacement of specific DNA fragments, to alter the sequence, expression levels or function of the target gene or regulatory element (Adiego-Perez et al. 2019; Arazoe et al. 2018). Double-strand breaks (DSBs) are DNA damage phenomena that occur when cells face certain adverse factors such as UV radiation or oxidative stress during natural growth and metabolic processes. Once a double-strand break occurs in the genome, cells utilize their own unique mechanisms to repair the lesion, mainly using HR or non-homologous end-joining (NHEJ) to avoid cell death (Sonoda et al. 2006). Prior to the introduction of genome editing technology, natural, physical, or chemical mutagenesis and random insertion of transgenic DNA were the main methods used to induce mutations in target cells. However, due to the randomness of DSBs, these methods cannot achieve genome editing at specific desired sites and are inefficient or costly (Blouzard et al. 2010; Mangan and Meijer 2001). Genome editing uses special engineered nucleases to introduce DSBs at specific locations in the genome and then achieves directed modifications to the genome through repair mechanisms, enabling metabolic pathway modifications, validation of biological functions of certain genes, or integration and expression of exogenous genes. CRISPR (Clustered Regularly Interspaced Short Palindromic Repeats) originating from the adaptive immune system in bacteria is a genome editing technology that depends on two key components: CRISPR-associated protein (Cas protein) and single-guide RNA (sgRNA) (Jinek et al. 2012). Currently, the most widely used CRISPR system is the type II CRISPR-Cas9 system. Cas9 is a nuclease that binds to sgRNA, and through a 20-bp nucleotide sequence present in the sgRNA, activates and directs Cas9 to a specific site in the genome (Protospacer Adjacent Motif, PAM site), where Cas9 subsequently cuts near the PAM site to produce a double-strand break in DNA. Then, the cell avoids death by using its own damage repair mechanisms (Doudna and Charpentier 2014). Since its discovery, the CRISPR-Cas9 system has been widely applied in the fields of animals, plants, and microorganisms due to its low cost, simplicity, and high efficiency. A study utilized the CRISPR-Cas9n system of a Cas9 protein mutant to achieve knockout of the yvmC gene in *B. licheniformis* (Li et al. 2018a). After the Cas9n cleaved the DNA single strand, integration of the target gene was accomplished by HR of complementary fragments. In this technology, the efficiency of HR is the rate-limiting step for genome editing. In our previous research, a knockout plasmid was constructed using a temperature-sensitive plasmid as a carrier to mediate homologous double-crossover to knock out the α-amylase gene. The resistance marker used for screening was later removed through FLP/FRT recombination (Li et al. 2019). This genome editing method still depends on the homologous recombination mechanism. Unfortunately, the recombination efficiency of *B. licheniformis* is quite low. This is a major obstacle for genetic manipulation and further industrial applications. The low efficiency of homologous recombination is mainly attributed to the lack of efficient recombinases. Therefore, the recombination efficiency is the key to the success of existing genome editing technologies.

Eukaryotes can directly repair breaks by NHEJ, whereas most prokaryotes including *B. licheniformis* lack the NHEJ system, and rely on HR systems to repair DSBs using a homologous sequence as a template. HR is ubiquitous in bacteriophages, bacteria, eukaryotes, and archaea (Vos and Didelot 2009). With the rapid development of molecular biology and genomics, various bacterial HR systems have been continuously discovered, and their recombination mechanisms have been intensively elucidated. The RecA recombination system is the first endogenous recombination system discovered in bacteria, originating from *E. coli*, and was discovered by screening for recombination-defective mutants. The system is catalyzed by a series of proteins including RecA, RecBCD, and RecFOR that have related auxiliary functions (Moody and Hayes 1972). Compared with that, recombinases identified in the Rac prophage of *Escherichia coli*, such as RecE and RecT, rely on recognition of homologous DNA sequences to achieve gene deletion or integration. RecE is an ExoVIII DNA endonuclease consisting of 866 amino acids with a protein molecular weight of 96.4 kDa. It can bind to and quickly cleave double-stranded DNA at a break point in the 5’-to-3’ direction, generating a 3’ overhang. RecT, on the other hand, is a 29.7 kDa single-stranded DNA annealing protein that can bind to both single-stranded and double-stranded DNA, mediating the invasion of linear homologous single-stranded or double-stranded DNA into supercoiled DNA (Thomason et al. 2016). The specific recombination mechanism of RecET recombinase involves the following steps. First, RecE with endonuclease activity cuts the double-stranded DNA molecule at the break point in the 5’-to-3’ direction, generating a 3’ overhang at the site of cleavage or degrading shorter substrates to single-stranded DNA. Subsequently, the single-stranded DNA annealing protein RecT binds to the degraded 3’ overhang or shorter single-stranded DNA to form a stable complex that prevents degradation of single-stranded DNA. Meanwhile, with the assistance of the RecT-DNA complex, RecT begins a genome-wide search for homologous sequences in double-stranded DNA as the elements for repairing breaks. Upon finding homologous DNA sequences, the two homologous segments form a Holliday junction structure according to the base pairing principle, ultimately achieving homologous recombination and repairing breaks (Castellanos and Romero 2009). It is worth noting that overexpression of RecT can significantly increase homologous recombination efficiency in the RecET recombination system, indicating that RecT is the main enzyme responsible for this process (Lloyd and Buckman 1995).

Since Murphy proposed an efficient genome editing system based on recombinases derived from phages in 1990 (Murphy et al. 1990), the use of phage-encoded recombinases has gained significant attention and has been widely adopted in molecular biology and genetic engineering studies. Promoters play a crucial role in regulating the expression of recombinases, allowing researchers to control the timing and level of recombinase production. Controlled expression of the recombinase genes is commonly achieved using promoters such as P_lac_, P_BAD_, or P_L_ promoter of phage λ (Ellermeier et al. 2002; Minorikawa and Nakayama 2011; Sauer and McDermott 2004). These promoters drive the expression of the recombinase genes, while also expressing the associated repressors / activators (lacI or araC) from the same plasmids. The promoters used for conditional expression of recombinant enzymes must meet the criteria of strictness and efficiency. Unfortunately, compared to *E. coli*, the availability of suitable conditionally-inducible promoters for *Bacillus* species, particularly *B. licheniformis*, is limited. Although the xylose-inducible expression system has been widely utilized in *Bacillus* species, it exhibits lower strictness (with a certain degree of background expression) while ensuring higher expression intensity (Li et al. 2018b). Therefore, it is necessary to explore and identify additional inducible expression promoters that meet the requirements for conditionally expressing recombinant enzymes in the construction of gene editing systems. Rhamnose-inducible promoters, such as the rhaBAD promoter derived from *E. coli*, have been extensively studied and optimized for controlled gene expression (Wilms et al. 2001). These promoters are tightly regulated in the absence of rhamnose, preventing background expression. Upon induction with rhamnose, they efficiently drive gene expression, enabling precise control over the timing and level of protein production. *B. licheniformis* also possesses the capability of metabolizing rhamnose. Investigating the functionality of endogenous rhamnose-responsive elements in this bacterium and employing them as conditional expression components holds great significance in optimizing the recombinases-based gene editing system of this strain.

In this study, we constructed a HR system using RecT recombinase from a bacteriophage with a conditional expression system driven by a native rhamnose promoter (P_rha_) in *B. licheniformis*. The genome editing efficiency was significantly improved by optimizing the conditions for RecT recombinase activity. This study provides a new genetic tool for engineering of *B. licheniformis*. It also has reference value for the development of genetic modification tools for other gram-positive bacteria.

## Materials and methods

### Media and strain cultivation

The bacterial strains, plasmids and primers used in this study are listed in Table 1. The reagents and medium for *Bacillus* transformation were prepared according to Li (Li et al. 2022). *E. coli* and *Bacillus* were grown in terrific broth (TB) or Luria-Bertani (LB) broth based on Xiao’s methods (Xiao et al. 2021). LBG and LBR media were formulated by incorporating 20 g/L glucose or 20 g/L rhamnose, respectively, into LB broth. 100 µg/mL ampicillin was added when necessary to maintain the plasmids in *E. coli*. *Bacillus* transformants were grown with 10 µg/mL erythromycin or 20 µg/mL tetracycline. Cultivation was performed at 37°C unless otherwise stated.

**Table 1:** Bacterial strains, plasmids and primers used in this study

Strain, plasmid or primers	Description or sequence (5’-3’)	Source or purpose
Strains		
*Escherichia coli* JM109	*F*', *traD36, proAB Δ. lacIq, Δ (lacZ), M15/Δ (lac-proAB), glnV44, e14*−, *gyrA96, recA1, relA1, endA1, thi, hsdR17*	Our lab
*Bacillus licheniformis* CICIM B1391	Wild-type	Our lab
BLA	*B. licheniformis* CICIM B1391 harboring plasmid pKA	This work
BLAR	*B. licheniformis* CICIM B1391 harboring plasmid pKAR	This work
BLAR2	*B. licheniformis* CICIM B1391 harboring plasmid pKAR2	This work
BLAR3	*B. licheniformis* CICIM B1391 harboring plasmid pKAR3	This work
BLAR4	*B. licheniformis* CICIM B1391 harboring plasmid pKAR4	This work
BLAR5	*B. licheniformis* CICIM B1391 harboring plasmid pKAR5	This work
BLKA	*B. licheniformis* CICIM B1391, *ΔamyL*	This work
BLPE	*B. licheniformis* CICIM B1391 harboring plasmid pHY300-P_rha_-eGFP	This work
Plasmids		
pMD19-T	*E. coli* cloning vector, Ap^R^	TaKaRa
pHY300-PLK	*E. coli*/*Bacillus* shuttle vector, Amp^r^ /Tet^r^	Our lab
pHY300-P_2_-eGFP	pHY, with the eGFP cassette mediated by P_2_ promoter	Our lab
pHY300-P_rha_-eGFP	pHY, with the eGFP cassette mediated by P_rha_ promoter	This work
pNZTT-AFKF	pNZTT, with the deletion cassette of *amyL*	Our lab
pKA	pHY-PLK300, with the deletion cassette of *amyL*	This work
pKAR	pKA, with the expression cassette of recombinase BPR1	This work
pKAR2	pKA, with the expression cassette of recombinase BPR2	This work
pKAR3	pKA, with the expression cassette of recombinase BPR3	This work
pKAR4	pKA, with the expression cassette of recombinase BPR4	This work
pKAR5	pKA, with the expression cassette of recombinase BPR5	This work
Primers		
*amyL*-*Hin*dIII-F	AAGCTTACGGCTTTATGCCCGATTGC	*amyL* deletion cassette
*amyL*-*Eco*RI-R	GAATTCCGATCCGCCGTTTACGTGAA	
P_rha_-*egfp*-F	AAAACGCTTTGCCCAAGCTTTCCTGACCCCTCCTTTTAAAAAACATGAG	*egfp* gene sequence
P_rha_-*egfp*-R	ACCATGGATCCGCGACCCATACGTATCACTCCGTTTTTGTTTGTTT	
P_rha_-R-1	TTTTTTCGTCGCCATACGTATCACTCCGTTTTTGTTTGTTT	BPR1 expression cassette
BPR1-F	AACGGAGTGATACGTATGGCGACGAAAAAACAAGAAGAAC	
BPR1-*Eco*RI-R	GAATTCTTATTCGTTGGTTTCGCCGC	
P_rha_-R-2	CCGGCCCGTATCCATACGTATCACTCCGTTTTTGTTTGTTT	BPR2 expression cassette
BPR2-F	AACGGAGTGATACGTATGGACACCGGCAGGAAG	
BPR2-*Eco*RI-R	GAATTCCTCGTTGGTCTCGCCGC	
P_rha_-R-3	TTTTTTCGTCGTCATACGTATCACTCCGTTTTTGTTTGTTT	BPR3 expression cassette
BPR3-F	AACGGAGTGATACGTATGACCACCAAGAAGCAGAGC	
BPR3-*Eco*RI-R	GAATTCCTCGTTGTCCTTCACCTCCAG	
P_rha_-R-4	TTCGTTTTTCGCCATACGTATCACTCCGTTTTTGTTTGTTT	BPR4 expression cassette
BPR4-F	AACGGAGTGATACGTATGGCCACCGAGAAGCAG	
BPR4-*Eco*RI-R	GAATTCCTGCTCGTCCACCACCTC	
P_rha_-R-5	TTTTTCCGTCGCCATACGTATCACTCCGTTTTTGTTTGTTT	BPR5 expression cassette
BPR5-F	AACGGAGTGATACGTATGGCCAAGAACGAGGACATCA	
BPR5-*Eco*RI-R	GAATTCGTCGAAGGGCAGGTCGTC	
*amyL*-YZ-F	CAGAAGCGGCGGAAGAGATT	Diagnostic PCR
*amyL*-YZ-R	ACGTTGCCATTTCATCCCCG	

### Plasmid construction

For construction of rhamnose-inducible expression plasmids, a fragment of the rhamnose promoter (P_rha_) was amplified via PCR technique with primers P_rha_-*egfp*-F and P_rha_-*egfp*-R, using the chromosomal DNA of CICIM B1391 as a template. Subsequently, DNA purification was performed using a Axygen Magnetic Beads DNA purification Kit (Corning, CA). The gene fragment of P_rha_ on the plasmid pHY300-P_2_-eGFP was excised using the restriction endonucleases *Hind*III and *Xho*I. After gel recovery purification, the linearized pHY300-eGFP vector was obtained. The P_rha_ fragment was then cloned into the linearized pHY300-eGFP vector via homologous recombination using a Axygen Magnetic Beads DNA purification Kit (Corning, CA) (Vazyme, China), following the instructions. The resulting product was transformed into *E. coli* JM109 competent cells, plated on ampicillin-resistant agar, and grown at 37℃ until single colonies were visible. Colony PCR was performed to verify the clones.

For construction of *amyL* deletion cassette, the *amyL* gene encoding an α-amylase (CP005965 REGION: 723302–724840) from *B. licheniformis* CICIM B1391 was chosen as the target gene for recombination and knockout experiments. Using the plasmid pNZTT-AFKF as a template, the knockout cassette fragment *amyL*-FRT-Kan-FRT-*amyL* was amplified using the primers *amyL*-*Hin*dIII-F and *amyL*-*Eco*RI-R, and the resulting PCR product was purified and digested with *Hin*dIII and *Eco*RI. The digested fragment was then ligated with the pHY300-PLK vector that had been double-digested with the same enzymes to construct the knockout plasmid pKA.

For construction of recombinase-mediated genome editing plasmids, the recombinant enzyme gene sequence was first synthesized by Sangon Biotech Co., Ltd (Shanghai, China). Take BPR1 as an example, the BPR1 gene fragment was amplified using the primers BPR1-*Xho*I-F and BPR1-*Eco*RI-R. The promoter sequence of the *rha* operon was amplified from *B. licheniformis* CICIM B1391 using the primers P_rha_-*Eco*RI-F and P_rha_-*Xho*I-R. The two fragments were then joined together by overlap extension PCR to generate the recombinant enzyme RecT expression cassette. After purification, the cassette was digested with *Eco*RI and ligated to the pKA plasmid that had been single-digested with the same enzyme to construct the knockout plasmid pKAR. Standard cloning and *E. coli* transformations were performed according to Sambrook and Russell (Sambrook et al. 2006). PCR reactions used PhantaTM Super-Fidelity DNA Polymerase from Vazyme biotech (Nanjing, China) and followed supplier instructions, primers were purchased from Sangon Biotech (Shanghai, China). All restriction enzymes were purchased from New England Biolabs (Ipswich, MA). Fermentas T4 DNA ligase were purchased through ThermoFisher Scientific (Waltham, MA). Vectors were isolated using an AxyPrep Plasmid Miniprep Kit from Axygen biosciences (Corning, CA).

### Fluorescence measurements

Overnight culture of the recombinant *B. licheniformis* was inoculate in to 30 mL of TB medium supplemented with 20 g/L glucose at an inoculation volume of 3%. This was then kept at 37℃ with orbital shaking of 250 rpm. 1 mL of culture was sampled at different time points. Cells were collected by centrifugation at 12 000 rpm, washed with 0.9% saline and diluted to OD_600_ = 0.5-1.0. GFP fluorescence was measured (SparK plate reader, Tecan, Männedorf, Switzerland) using a 96-well microtiter plate at an excitation wavelength of 485 nm, emission wavelength of 535 ± 15 nm and a gain value of 100. Average fluorescence and standard deviation were calculated from the geometric mean fluorescence values of technical triplicates.

### Recombinase conditional expression-mediated gene knockout

The constructed knockout plasmids pKA and pKAR were separately transformed into *B. licheniformis* CICIM B1391 to obtain strains BLA and BLAR by electroporation, according to the method described by Zhang et al. (Zhang et al. 2021), with a minimum plasmid concentration of 200 ng/µL. The transformed BLA and BLAR strains were transferred to 15 mL LB medium supplemented with tetracycline and grown at 30℃ and 200 rpm. After induction with rhamnose to promote P_rha_ expression, the cultures were further incubated for a certain period. Diluted culture aliquots were plated on kanamycin-containing plates and incubated at 37℃ until single colonies emerged. Simultaneously, 500 µL of the culture was transferred to fresh 15 mL LB medium supplemented with tetracycline under the same conditions for subculture, and the diluted culture was plated on the same type of plates. A few single colonies were picked, and PCR was performed using knockout validation primers *amyL*-YZ-F/*amyL*-YZ-R to confirm the knockout.

### HPLC detection for rhamnose

The culture of recombinant strains was centrifuged at 12,000 rpm for 1 minute, and 300 µL of the supernatant was collected. Then, 200 µL of the supernatant was mixed with an equal volume of 10% trichloroacetic acid solution and incubated at 4℃ for 3 hours. After centrifugation at 12,000 rpm for 20 minutes, the supernatant was collected and passed through a membrane filter with a 0.2 micron pore size. For chromatography, a Dikma CarboPac H^+^ sugar column was used, with 0.5‰ diluted sulfuric acid as the mobile phase at a flow rate of 0.80 mL/min and a column temperature of 50℃.

### α-amylase Assay

Shake flask fermentation of wild-type and knock-out strains was performed in TB medium at 37°C and 250 rpm for 24 hours. The bacterial cultures were then centrifuged, and the resulting supernatant was collected as the crude α-amylase enzyme solution. To measure the enzyme activity, 200 µL of the solution was added to 800 µL of 1% (w/v) soluble starch solution preheated at 40°C for 5 minutes. The mixture was then incubated at 40°C for 30 minutes with shaking. After incubation, 500 µL of the reaction solution was transferred to a colorimetric tube, and 1.5 mL of dinitrosalicylic acid (DNS) reagent was added. The mixture was then heated in boiling water for 5 minutes, and the volume was made up to 25 mL with deionized water. The absorbance was measured at 540 nm (Li et al. 2020). The enzyme activity was calculated based on the amount of reducing sugar generated. One unit of α-amylase activity (U) was defined as the amount of enzyme required to hydrolyze soluble starch and produce 1 µmol of maltose per hour under the above reaction conditions.

## Results

### Genome mining of the recombinases

Studies have shown that the recombination system derived from *Bacillus* and its bacteriophages can efficiently edit bacterial genomes, mainly mediated by the RecT family of proteins. The recombination system mediated by the RecE/RecT recombinases has been successfully applied to gene editing in various microorganisms (Lo Piano et al. 2011; Xin et al. 2017). Recombinases have been found to have host preferences, where those derived from natural or related bacteriophages can significantly enhance host recombination frequency (Smith and Dorman 1999; Vellani and Myers 2003). Similarly, the RecT recombinase usually performs well in its native or closely related microbial host. Therefore, we first retrieved a putative Rec-T recombinase, QFR56352.1, originating from *Bacillus* phage, from the NCBI database and designated it as BPR1. Subsequently, using its amino acid sequence as a template, we conducted further searches and obtained four additional putative Rec-T recombinases, named BPR2-5. These sequences exhibited varying degrees of similarity to BPR1 at the amino acid level (Table 2 and Supplementary Fig. 1a). The homology between BPR1 and BPR2 was 84.67%, with BPR3 was 61.54%, with BPR4 was 54.55%, and with BPR5 was 41.37%. Sequence homology implies functional similarity of these proteins, and thus, we predicted that all five recombinases have homologous recombination activity in *Bacillus*. We constructed a phylogenetic tree of the recombinases using MEGA 7.0 neighbor-joining method (Supplementary Fig. 1b), which indicated that the recombinases had certain distance in their evolutionary relationships. Exploring these amino acid sequences with varying degrees of similarity broadens the scope of recombinases mining and facilitates the development these enzymes with improved functionality for various applications.

**Table 2:** Information of the five recombinases

Name	GenBank accession	Source
BPR1	QFR56352.1	*Bacillus* phage 049ML001
BPR2	QEG13505.1	*Bacillus* phage vB_BspS_SplendidRed
BPR3	YP_009010510.1	*Geobacillus* phage GBK2
BPR4	QIQ61250.1	*Bacillus* phage vB_BcM_Sam46
BPR5	WP_115997067.1	*Bacillus amyloliquefaciens* phage

### Development of a conditional expression system in response to rhamnose

**Fig. 1:**
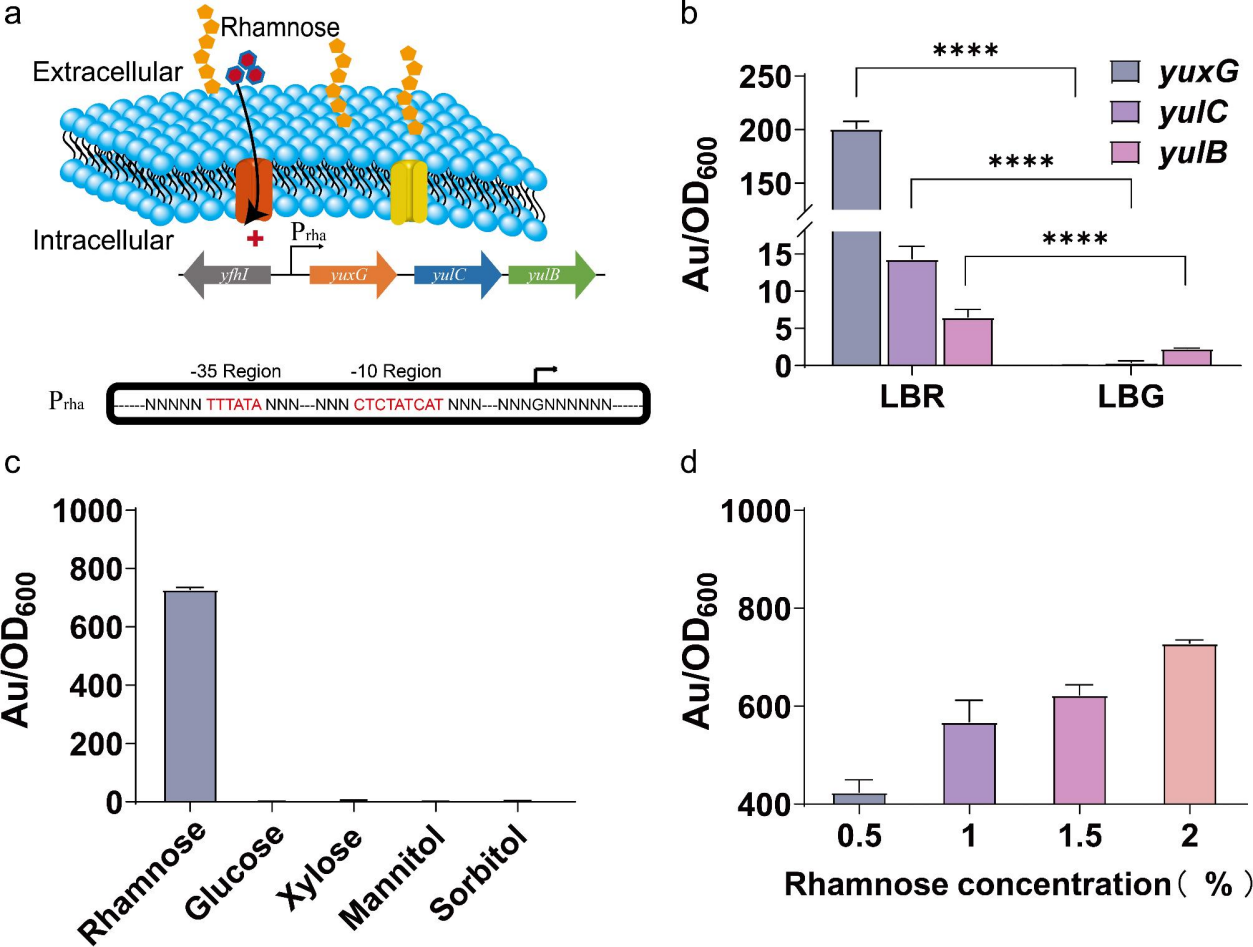
Identification and characterization of a conditional expression system in response to rhamnose. Prediction of the promoter core sequences in the operon (**a**). The relative expression levels of the genes in LBR and LBG media (**b**). Relative fluorescence intensity of the recombinant strains grown in different carbon sources (**c**). Relative fluorescence intensity of the recombinant strains grown in media with different concentrations of rhamnose (**d**)

Through analysis of NCBI data, the endogenous rhamnose metabolic pathway of *B. licheniformis* was discovered. By searching for the upstream and downstream genes of the rhamnose metabolic pathway, a gene cluster was identified. Upstream of this gene cluster is the efflux transporter synthesis gene *yfh*I, with a 359 bp interval between it and the rhamnose metabolic gene cluster, which was predicted as a promoter sequence and named P_rha_. This gene cluster contains three putative genes for rhamnose catabolism: rhamoral aldolase gene *yux*G (sequence number: AGN37940.1), rhamnose kinase gene *yul*C (sequence number: AGN37938.1), and its transcriptional regulator gene *yul*B (sequence number: AGN37936.1). The sequence of this gene cluster was compared with that of *B. subtilis*, showing that *yux*G has 75.76% similarity to *rha*E, *yul*C has 38.34% similarity to *rha*B, and *yul*B has 69.38% similarity to *rha*R (Hirooka et al. 2016). Based on the above analysis, it is speculated that this gene cluster is a rhamnose operon in *B. licheniformis*. The rhamnose promoter gene sequence was also submitted to BPROM (http://www.softberry.com/) for prediction of the core sequence (Anzolini Cassiano and Silva-Rocha 2020). The predicted promoter structure is shown in Fig. 1a, with the − 35 region being TTTATA and the − 10 region being CTCTATCAT.

Next, total RNA was extracted from the culture of *B. licheniformis* in LBR and LBG media after 24 h, respectively. As shown in Supplementary Fig. 2, the bands of 23 S rRNA, 16 S rRNA, and 5 S rRNA for all three genes in LBR and LBG media were clear, indicating good RNA extraction efficiency. The relative expression levels (fluorescence intensity (AU)/OD_600_) of the genes in the rhamnose operon were then measured using RT-qPCR, with the housekeeping gene *rsp*E used as the internal reference gene. In LBR medium, the relative expression levels of the three related genes in rhamnose operon, *yux*G, *yul*C, and *yul*B, were 200.92, 14.25, and 6.55, respectively, suggesting that the transcription levels of the three genes were significantly up-regulated upon rhamnose addition. In contrast, in LBG medium, the relative expression levels were 0.045, 0.39, and 2.33, respectively, with minimal changes observed (Fig. 1b). These results indicated that the expression of *yux*G, *yul*C, and *yul*B genes are regulated by P_rha_. Furthermore, a recombinant plasmid was constructed to investigate the transcriptional expression characteristics of P_rha_ with an enhanced green fluorescent protein gene (eGFP) as a reporter gene, to yield BLPE. The strain was cultivated utilizing five distinct carbon sources, namely glucose, sorbitol, mannitol, xylose, and rhamnose, as the exclusive carbon source at a standardized concentration of 20 g/L. Results showed that the strain can fully utilize five carbon sources within 48 h. Although there was no significant difference in the final bacterial cell density, there were noticeable variations in fluorescence intensity. Specifically, a relative fluorescence intensity of the strain grown in rhamnose was over 700, whereas that was barely detected when glucose, sorbitol, mannitol, or glycerol were used as the sole carbon source (Fig. 1c). This indicates that P_rha_ can only initiate *eGFP* gene expression and produce green fluorescence when rhamnose is used as the sole carbon source. It can also be concluded that this promoter is strictly induced by rhamnose. Finally, BLPE was cultured in media with different initial concentrations of rhamnose (0.5%, 1%, 1.5%, and 2%) for 24 h. As shown in Fig. 1d, the relative fluorescence intensity increased with an increase in rhamnose concentration, indicating that the expression level of the target gene can be regulated by the concentration of rhamnose.

### Construction of *amyl*-deleting plasmids and genome editing

The construction of the *amyL* gene knockout plasmid is shown in Supplementary Fig. 3a. The plasmid pKA, containing the *amyL* knockout cassette, was verified by double digestion with *Hin*dIII and *Eco*RI. The theoretical size of the pHY300-PLK vector is 4840 bp, and the size of the *amyL* knockout cassette fragment is 2203 bp. The plasmid pKAR1-5, containing the RecT expression cassette, was verified by single digestion with *Eco*RI. The theoretical size of the pKA vector is approximately 7000 bp, and the size of the RecT expression cassette fragment is approximately 1200 bp. The sizes of the digested fragments of both plasmids were consistent with the theoretical sizes (Supplementary Fig. 3b,c), indicating successful construction of the knockout plasmids pKA and pKAR1-5.

**Fig. 2:**
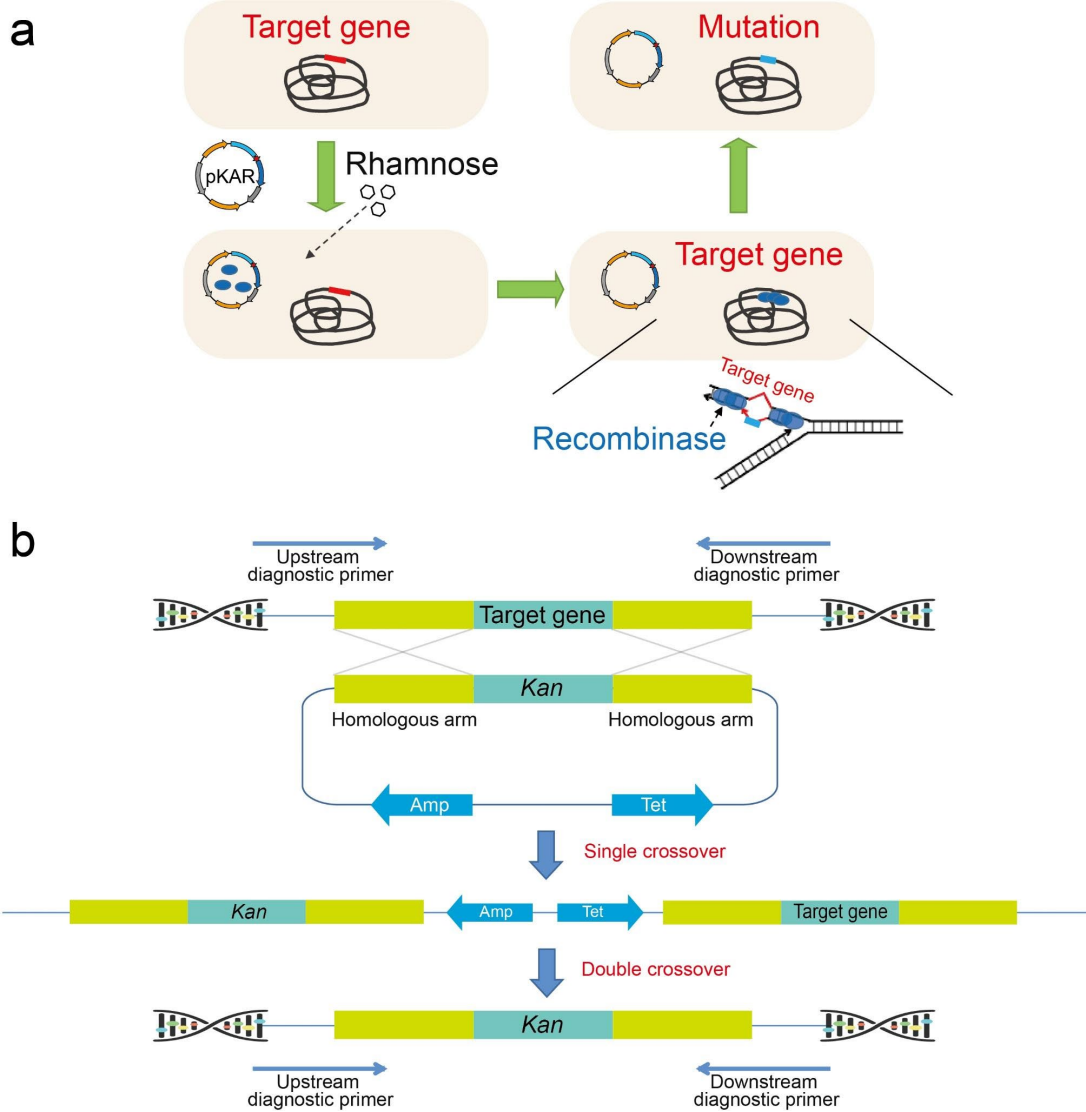
Editing of target gene in the *B. licheniformis* chromosome using a controllable recombinase system (**a**). Construction scheme of the genome editing plasmid and the method for transformants verification (**b**)

The gene editing strategy of the recombinant system constructed in this study is shown in Fig. 2a. The knockout plasmid pKAR was transformed into *B. licheniformis* to obtain the recombinant strain BLAR, which was then cultured at 37°C and 200 rpm in a shaker flask. During the culture of the recombinant strain, 1% rhamnose was added to induce the expression of the recombinase RecT. After that, the RecT recombinase binds to DNA double-strand breaks and exerts its genome-wide search function to find homologous segments carried on the pKAR plasmid. Recombination occurs with a certain probability during the shaker flask culture, and mutant strains are screened by diluting the bacterial solution at a suitable time and spreading it on a kanamycin resistance plate for colony PCR. In the process of strain cultivation, both homologous arms undergo recombination to insert the artificial segment into the target site on the genome, completing the double exchange and successful homologous recombination (Fig. 2b). Finally, the colony PCR primers were designed upstream and downstream of the target gene in the transformants’ genome.

**Fig. 3:**
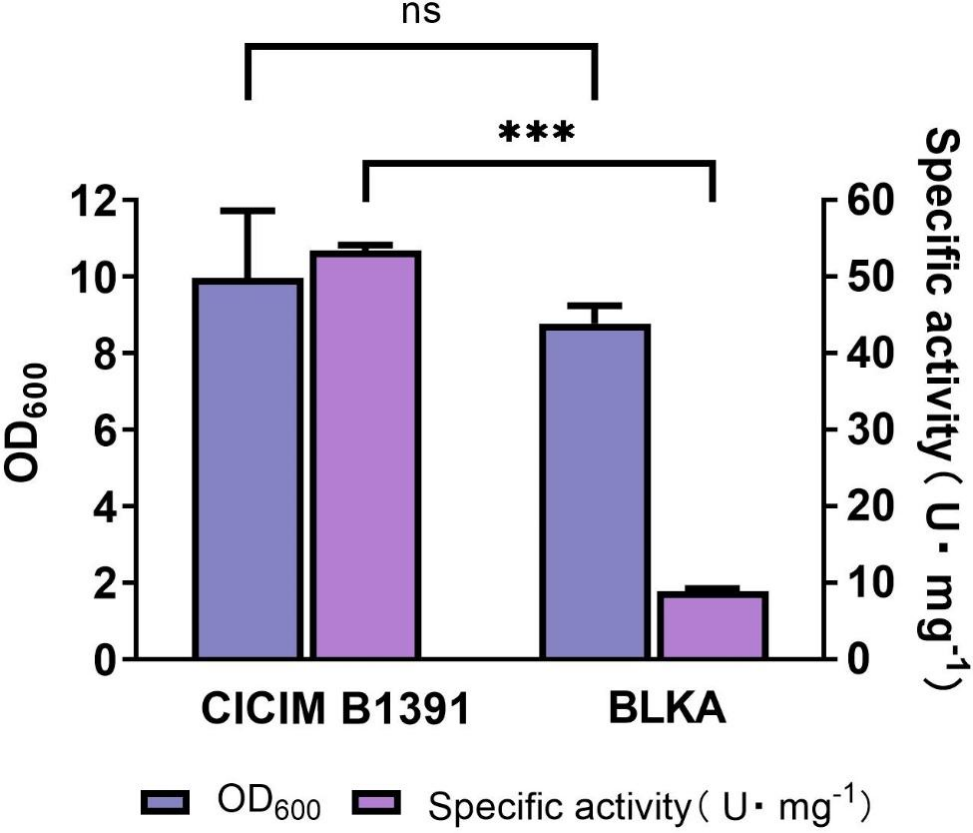
Partial diagnostic PCR results of the transformants obtained through genome editing using BPR1 are presented (**a**). Transformants with an edited genetype exhibit a distinct band of 2649 bp, while those with a wild-type genetype display a band of 1947 bp. The recombination effi-ciency was evaluated by cultivating the BLAR strain in media supplemented with rhamnose (BLAR-Rha) or without rhamnose (BLAR), with the wild-type strain BLA serving as a control (**b**)

The experimental results showed that among the five recombinases used in the constructed recombinase system, only BPR1 displayed high recombinase activity and successfully generated positive transformants with gene knockout (Partial diagnostic PCR results are presented in Fig. 3a), with a calculated recombination efficiency of 5.56%. As a control, the recombinant strains without recombinase and the recombinant strains without induction of rhamnose promoter (P_rha_) did not generate positive transformants with gene knockout (Fig. 3b). The other four recombinases did not demonstrate recombination potential, and no positive transformants were screened from the BLAR2-4 recombinant strains. These results demonstrate that the strategy of regulating the expression of recombinase RecT with the P_rha_ promoter is effective, and the RecT recombinase from *Bacillus* phage 049ML001 exhibits high recombination activity in *B. licheniformis*, indicating that the homologous recombination system constructed using it can successfully knock out the *amyL* gene in this strain.

### Plasmid curing and resistance recovery

**Fig. 4:**
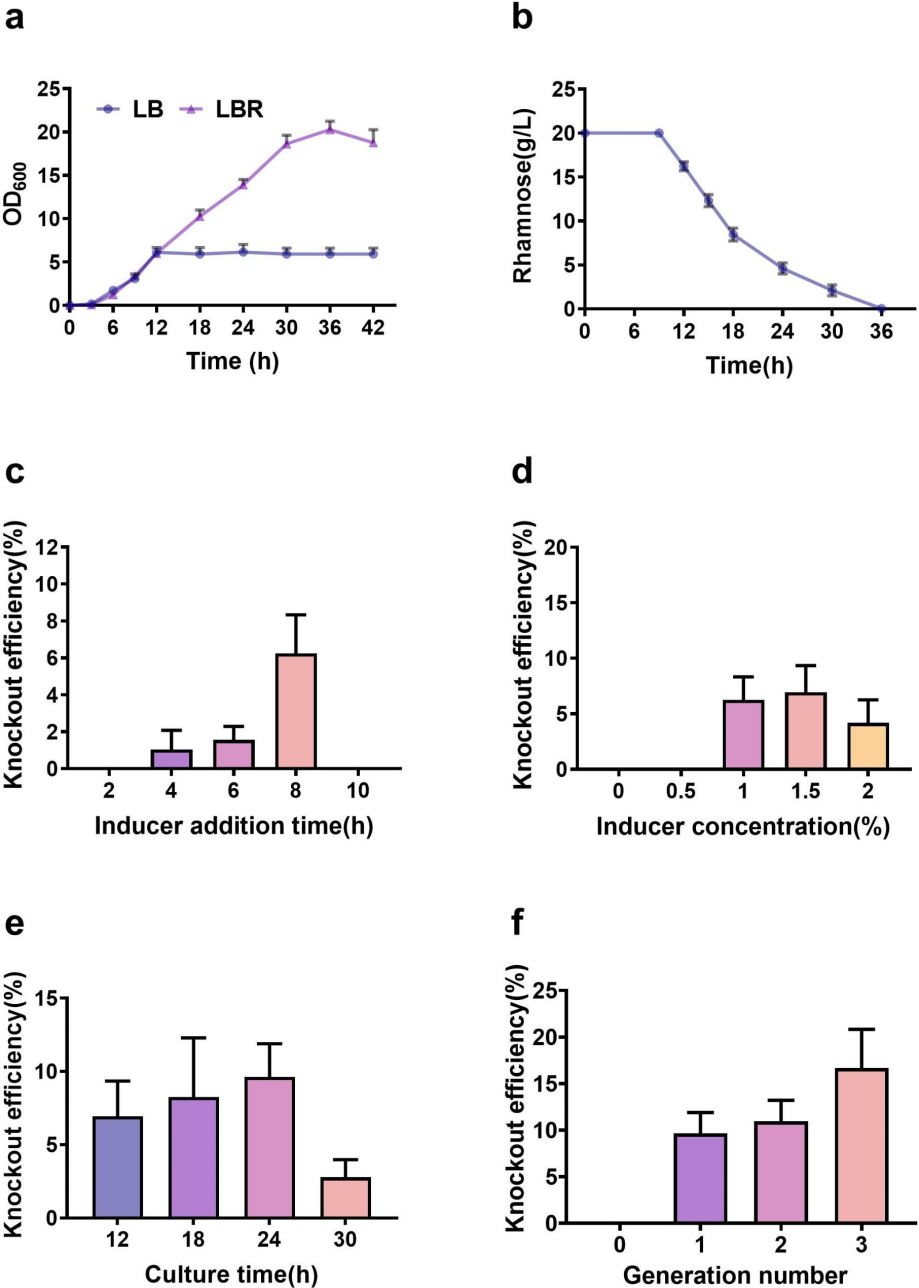
Plate screening of the recombinants (**a**). Plasmid curing efficiency of transformants with different generations of propagation (**b**). PCR verification of the screened transformants using primers *amyL*-YZ-F and *amyL*-YZ-R (**c**)

Plasmid curing has a significant impact on the efficiency of genome editing. On one hand, plasmid curing can enhance the efficiency of genome editing by eliminating potential interference or competition between the gene editing plasmid and endogenous plasmids within the bacterial host. This can lead to improved transformation efficiency and increased stability of the desired genetic modifications. On the other hand, plasmid curing can also reduce the efficiency of genome editing. The loss of the gene editing plasmid may result in the loss of essential components or genes required for the editing process. This can hinder the ability to introduce specific genetic modifications or disrupt the desired editing outcomes (Wang et al. 2021). Therefore, we investigated the process of plasmid curing in B. licheniformis to evaluate the feasibility of iterative genome editing by reusing this genome editing plasmid. After successfully knocking out the *amyL* gene, positive transformants were picked and inoculated into 15 mL LB medium without antibiotics at 37℃ and 200 rpm to lose plasmid pKAR. After 12 h of non-antibiotic cultivation for each generation of bacteria, the bacterial liquid was diluted and spread on kanamycin-resistant plates. Thirty-two single colonies were selected and streaked on tetracycline-resistant plates one by one. The plasmid pKAR curing efficiency was calculated according to the sensitivity of single colonies to tetracycline resistance. Some streaking results are shown in Fig. 4a. Following a single generation of non-antibiotic cultivation, one out of the 32 individual colonies exhibited sensitivity to tetracycline resistance. Through multiple parallel experiments, the plasmid curing efficiency was determined to be 1.56% at this stage. Subsequently, after two generations of non-antibiotic cultivation, the plasmid curing efficiency increased to 17.19%. Further non-antibiotic cultivation for three generations resulted in a plasmid curing efficiency of 31.25%. Notably, after four generations of non-antibiotic cultivation, the plasmid curing efficiency reached 48.44%, approaching a half (Fig. 4b). The above results indicate that non-antibiotic passage culture can lose plasmids carried by pHY300-PLK vector, and with the increase of passage times, the plasmid curing efficiency gradually increases.

Although the *amyL* gene of the strain has been inactivated by deletion and insertion mutations through the recombination system and the knockout plasmid has been lost, resistance genes still remain on the genome, and the strain carries kanamycin resistance. Li’s method was used to delete and recover the resistance marker using the anti-resistance plasmid pNZTT-flp (Li et al. 2020). PCR verification was performed using primers *amyL*-YZ-F and *amyL*-YZ-R. The results are shown in Fig. 4c. After resistance recovery, the band size was 1463 bp, which is significantly smaller than that of the original strain (1947 bp) and before resistance recovery (2649 bp), indicating that resistance recovery was successful.

### α-amylase assay for *amyL* knockout strains

**Fig. 5:**
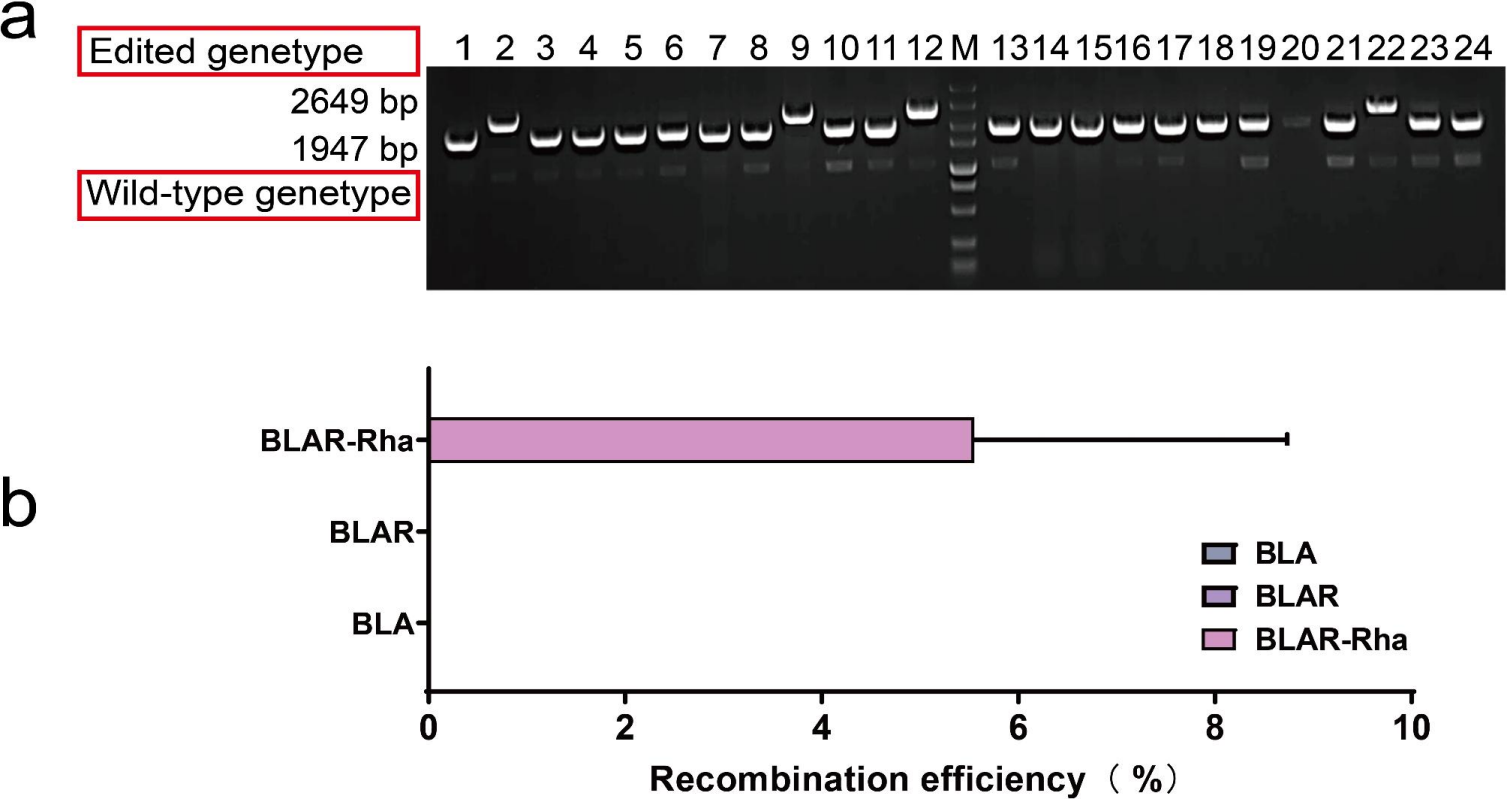
Comparison of the cell growth and specific α-amylase activity between the wild-type strain (CICIM B1391) and the edited one (BLKA)

After verifying the knockout of *amyL* at the molecular level, in order to further verify the knockout effect of the recombinant system, α-amylase activity assay was carried out on the wild-type strain B1391 and the *amyL* knockout strain BLKA. The results are shown in Fig. 5. The specific α-amylase activity of BLKA culture was determined to be 8.86 U·mg^-1^, which was reduced by 83.41% compared with that of B1391 (53.42 U·mg^-1^). This indicates that knocking out the *amyL* gene greatly reduces the extracellular amylase production of *B. licheniformis*. Moreover, it can be seen from bacterial cell growth of both strains in a medium without starch substances, knocking out the *amyL* gene will not cause significant negative effects on cell growth. This is consistent with published research results, which show that starch coding genes are dispensable for cell growth, especially for *Bacillus* strains that usually have complex carbohydrate hydrolysis enzyme systems (Li et al. 2020). In summary, the above molecular level and enzyme activity level verification results all indicate that using recombinant enzyme BPR1 to construct a recombinant system can be applied to gene knockout without causing negative effects on bacterial growth and is a promising gene editing system.

### Optimization of the conditional gene-knockout

In order to investigate further the factors affecting the efficiency of gene editing, the recombination system was optimized by changing the induction time, the concentration of the inducer, the propagation time of the strain and the generation number of propagation, in order to improve the efficiency of gene editing.

**Fig. 6:**
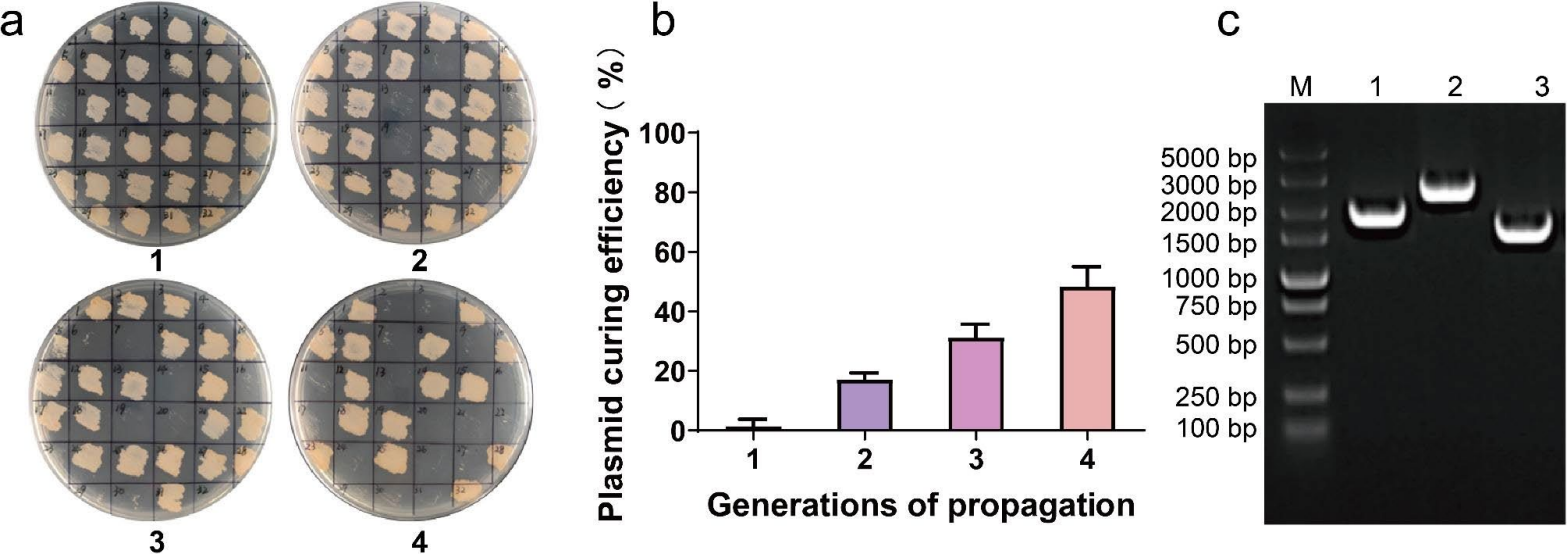
The effect of different conditions on the efficiency of gene-knockout. Cell growth in LB and LBR media (**a**). Rhamnose could be utilized in 36 h (**b**). The effect of different inducer addition times on the efficiency of gene-knockout (**c**). The effect of different inducer concentrations on the efficiency of gene-knockout (**d**). The effect of different culture times on the efficiency of gene-knockout (**e**). The effect of different generation numbers on the efficiency of gene-knockout (**f**)

As the rhamnose-inducible promoter P_rha_ is not yet fully understood in terms of its regulation of the expression of the recombinase RecT, the addition time of rhamnose was investigated at 2, 4, 6, 8, and 10 hours after inoculation to observe the gene knockout efficiency. When rhamnose was added at 8 hours after inoculation, the highest number of positive transformants was obtained, and the knockout efficiency was calculated to be 6.25% (Fig. 6c). Combined with the growth curve of the recombinant strain BLAR in the LB and LBR media, as well as the rhamnose consumption curve (Fig. 6a,b), it was found that the biomass of BLAR in the LBR medium was lower than that in the LB medium within 9 hours. The strain began to consume rhamnose after hours of lag phase. The biomass of the BLAR began to increase logarithmically, far exceeding that in the LB medium. Based on these results, it was speculated that rhamnose at a specific concentration may have a slight inhibitory effect on cell growth of the strain. Therefore, adding rhamnose after enriching the biomass of the strain BLAR for 8 hours of growth is more effective in inducing the expression of the recombinase. This condition is beneficial for improving the efficiency of gene knockout.

Different concentrations of rhamnose were added to investigate the effect of the inducer concentration on the activity of the recombinase. After 8 h of growth, rhamnose was added at concentrations of 0.5%, 1%, 1.5%, and 2% to initiate the expression of RecT gene. When the concentration was 0.5% or lower, no positive transformants were obtained, suggesting that the transcriptional activity of the P_rha_ promoter was too low, resulting in a low expression intensity of the recombinase. Gene editing could hardly achieved. The highest number of positive transformants was obtained when the inducer concentration was 1.5%, and the recombination efficiency was calculated to be 6.94% (Fig. 6d). However, when the concentration of the inducer was increased to 2%, the gene editing efficiency slightly decreased. This could because when the RecT functions in the bacterial cells, it influences both metabolic processes and genome stability. Overactivity of the recombinase can disrupt the intracellular balance and have harmful effects on the cells, even leading to abnormal elimination of genome fragments and causing genetic damage. Therefore, the expression of the RecT recombinase needs to be controlled at an appropriate level. Thus, the concentration of rhamnose needs to be selected accordingly to maintain the expression intensity of RecT recombinase, at which the efficiency of gene editing can be maximized.

The propagation time for recombinant strains was compared between 12, 18, 24, 30, and 36 h. The highest number of positive transformants was obtained after 24 h of induction, with a gene knockout efficiency of 9.64% (Fig. 6e). During fermentation of BLAR in LBR medium, rhamnose consumption lasted for 27 h, which was much slower than the utilization of glucose and other carbon sources (Fig. 6b). It was speculated that the slow utilization of rhamnose was beneficial for the sustained and stable expression and function of the recombinase. Therefore, appropriately prolonging the culture time can improve the gene knockout efficiency. In addition, related research results showed that when rhamnose was used to regulate the expression of exogenous genes in *B. subtilis*, the rhamnose promoter had a more significant induction effect in the late logarithmic growth phase (Hirooka and Tamano 2018). However, with the prolonged culture time of the strain in the logarithmic growth phase, there was a decrease in the induction rate of rhamnose. Consequently, an excessively long culture time is not favorable for the induction effect of the rhamnose promoter and may even result in a reduction in the number of positive transformants. Therefore, it is crucial to control the propagation time of the strain in the late logarithmic growth phase to achieve the highest efficiency in gene editing, following a 24-hour induction period.

To investigate the effect of different generation numbers of propagation on the recombination efficiency, the strain was continuously subcultured. The results showed that the highest gene knockout efficiency (16.67%) was achieved after three generations (Fig. 6f). During homologous recombination, single exchange events can occur, and the strains with single exchange can exhibit two tendencies during continued culture: one is to generate revertant mutations and restore the original genotype, while the other is to complete double exchange, resulting in gene knockout or knock-in. With an increase in generation times, the probability of double exchange strains under the sustained action of the recombinase RecT increases. Therefore, increasing the number of passages can improve the gene knockout efficiency. However, considering the time and cost of the experiment and the increased risk of contamination by foreign bacteria during multiple passages, a suitable number of passages should be chosen.

## Discussion

Recombineering using phage-encoded recombinases, was first introduced as an *in vivo* genetic engineering tool for *E. coli* and its genetically related species by Murphy in 1990 (Murphy et al. 1990). Recombinases from *Bacillus* phages have been shown to have high recombination efficiency and specificity, making them attractive tools for genetic engineering applications. Comparison of recombineering systems from different origins apparently suggested that host-specific factors constrained their application in genetically distant species (Bouchard and Moineau 2000; Datta et al. 2006). Fortunately, various annealing proteins, such as RecT-like and Redβ-like proteins, have been identified by genome mining, providing convenience for researchers to expand recombineering beyond module organisms to more distantly related bacterial strains (Datta et al. 2008; Dong et al. 2014; Zhang et al. 1998). *B. licheniformis* is an important industrial microorganism mainly producing its native enzymes or metabolites. The extremely low transformation efficiency undermines successful genetic manipulation in *B. licheniformis*, even if the same method is easily applied in other genera when the genetic background of the source host is well known. The traditional homologous recombination technique in this strain relies on the bacterial endogenous recombination system, which is a self-repair mechanism that bacteria use to cope with external environmental pressures. However, considering the low transformation efficiency, the traditional homologous recombination technique hardly contributes to genome editing in this strain. Therefore, five RecT recombinases with both homology and diversity were identified and introduced into *B. licheniformis* as a heterologous recombination system. Using a new gene editing strategy, the α-amylase *amyL* gene was knocked out, and it was determined that the RecT recombinase from *Bacillus* phage 049ML001 has the most significant recombination potential in *B. licheniformis*. The recombinase-based recombination system can successfully knock out the *amyL* gene without causing a significant negative impact on bacterial growth. Therefore, the recombinase-based system is an effective gene editing tool that can be applied to *B. licheniformis* for gene knockout. Meanwhile, the heterologous recombination system avoids the strict regulation of gene recombination in bacteria, and the recombination efficiency is 10^5^ times higher than that of the endogenous recombination efficiency of *B. licheniformis*, which is reported around 10^− 6^ (Waldeck et al. 2006).

A common issue encountered in the application of recombinases is that its expression can have toxic effects on bacterial cell growth, particularly if it is expressed at high levels or for prolonged periods of time (Akboga et al. 2022). One possible mechanism of toxicity is that recombinase-mediated recombination can cause DNA damage or rearrangements, which can lead to chromosomal instability, cell death, or growth inhibition. Recombinase expression can also interfere with essential cellular processes such as DNA replication, transcription, and translation, leading to cell stress and reduced viability. Another possible mechanism is that recombinase expression can cause metabolic imbalances or depletion of critical cellular resources. For example, the overexpression of certain recombinases may require high levels of ATP or other energy sources, which can impair cellular respiration and growth (Wang et al. 2019). Recombinase expression may also lead to the depletion of essential cofactors or substrates, such as nucleotides or amino acids, which can impair the synthesis of cellular macromolecules and disrupt normal metabolic pathways. Controlling the expression of recombinase with an inducible promoter is a strategy to mitigate the toxic effects, however, the type and strength range of the inducible promoter needs to be carefully selected. We have found that *B. licheniformis* can use rhamnose as the sole carbon source for growth, indicating that this strain contains rhamnose utilizing genes. Three genes, *yuxG*, *yulC*, and *yulB*, were predicted to be involved in rhamnose metabolism, and their expression was governed by a promoter P_rha_. When rhamnose is present outside the cell, the transporter protein harbors rhamnose into the bacterial cell. Then rhamnose is phosphorylated and broken down. YulB and CcpA (a global transcription factor that controls the degradation of multiple carbon metabolites) together regulate P_rha_, activating the transcription of the rhamnose gene cluster (Xiao et al. 2021). By observing the effects of different carbon sources on the regulation of green fluorescent protein expression by promoters, it was demonstrated that the P_rha_ is a strictly rhamnose-inducible promoter. Therefore, the influence of recombinase expression on cell growth can be controlled by rhamnose supplementation.

Through the evaluation and optimization of induction conditions, our study demonstrated that a high recombineering efficiency can be achieved in *B. licheniformis*. When the cell concentration reaches the desired threshold, the addition of the inducer initiates the biosynthesis stage, and the promoter regulates the expression of the relevant genes. Moreover, the expression level is positive correlated to the inducer concentration. Therefore, by changing the time and concentration of the addition of rhamnose, a suitable induction time and gene expression intensity can be obtained, leading to an improvement in the gene knockout efficiency of the recombination system. Further improvement of the gene editing efficiency of the system was achieved by varying the propagation time and generation number. Ultimately, it was determined that the highest gene editing efficiency of up to 16.67% could be achieved by inducing the expression of the recombinase RecT by adding 1.5% rhamnose 8 hours after growth and continuing to culture for 24 hours with three passages after knocking out the α-amylase gene *amyL*.

In conclusion, our study provides insights into the functionality of bacterial phage originated RecT recombinase in *B. licheniformis* and demonstrates the successful application of recombineering systems in gene knockout. Future work is needed to further improve the recombination efficiencies of these systems in other *Bacillus* species and to adapt them to other sequenced strains of the genus, including the strains used as industrial producers and in health applications.

### Electronic supplementary material

Below is the link to the electronic supplementary material.

**Supplementary Figure 1**. Amino acid sequence alignment of the putative recombinases (a). Phylogenetic tree of the recombinases (b). **Supplementary Figure 2**. Total RNA extracted from B. licheniformis cultured in LBG (a) and LBR (b) media. **Supplementary Figure 3**. Construction scheme of genome editing plasmid pKAR (a). Gel analysis of the plasmid pKA digested by EcoRI and HindIII (b). Gel analysis of the plasmid pKAR1-5 digested by EcoRI (c).
