# Supplementary material for: Construction of a bacteriophage-derived recombinase system in Bacillus licheniformis for gene deletion
Source: AMB Express. 2023 Aug 26;13:89. doi: 10.1186/s13568-023-01589-w (PMC10460339; doi:10.1186/s13568-023-01589-w)
Supplement: Supplementary file 1 — Supplementary Figure 1. Amino acid sequence alignment of the putative recombinases (a). Phylogenetic tree of the recombinases (b). Supplementary Figure 2. Total RNA extracted from B. licheniformis cultured in LBG (a) and LBR (b) media. Supplementary Figure 3. Construction scheme of genome editing plasmid pKAR (a). Gel analysis of the plasmid pKA digested by EcoRI and HindIII (b). Gel analysis of the plasmid pKAR1-5 digested by EcoRI (c). [file 13568_2023_1589_MOESM1_ESM.docx]

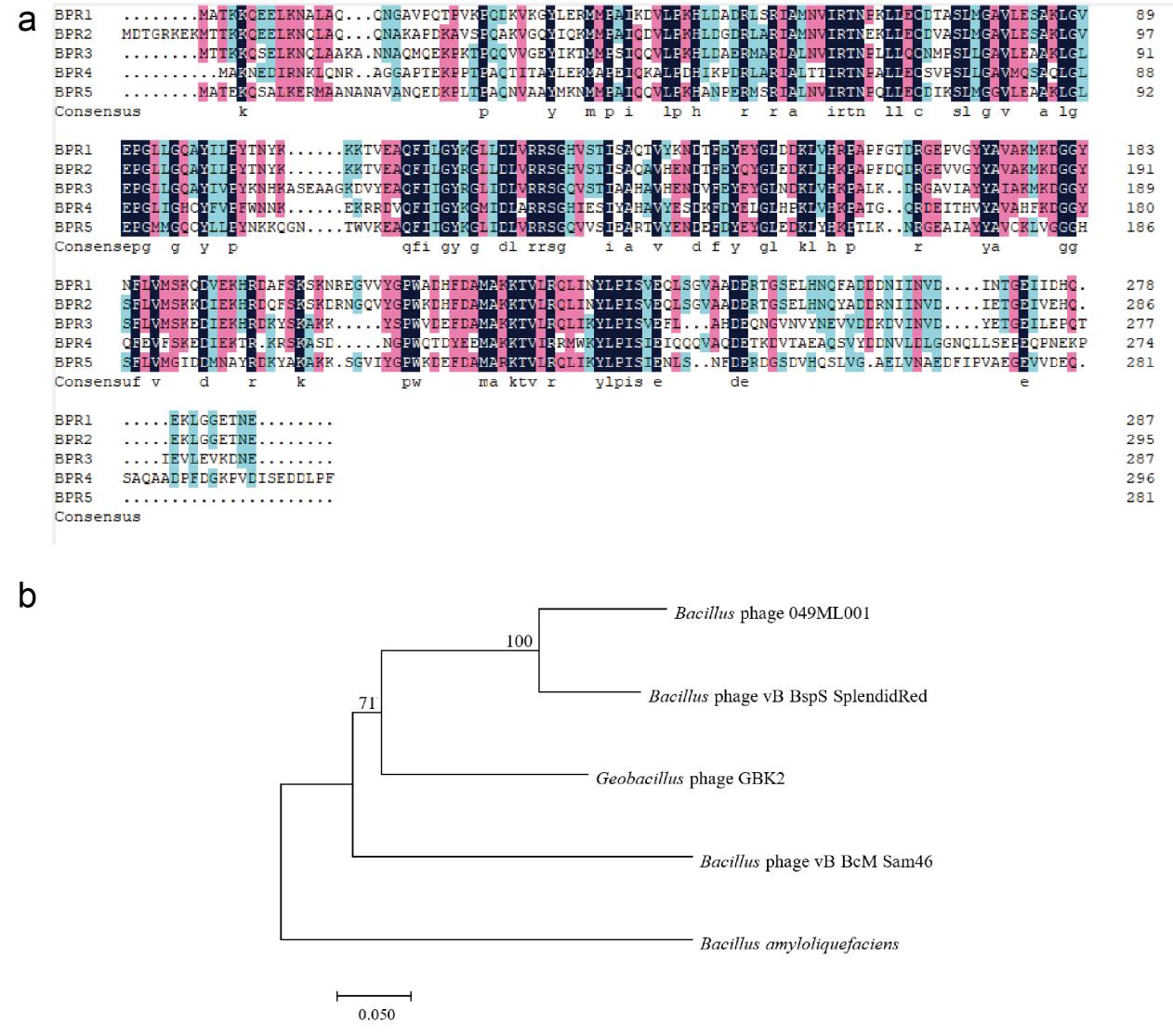


**Supplementary Figure 1.** Amino acid sequence alignment of the putative recombinases (a). Phylogenetic tree of the recombinases (b).


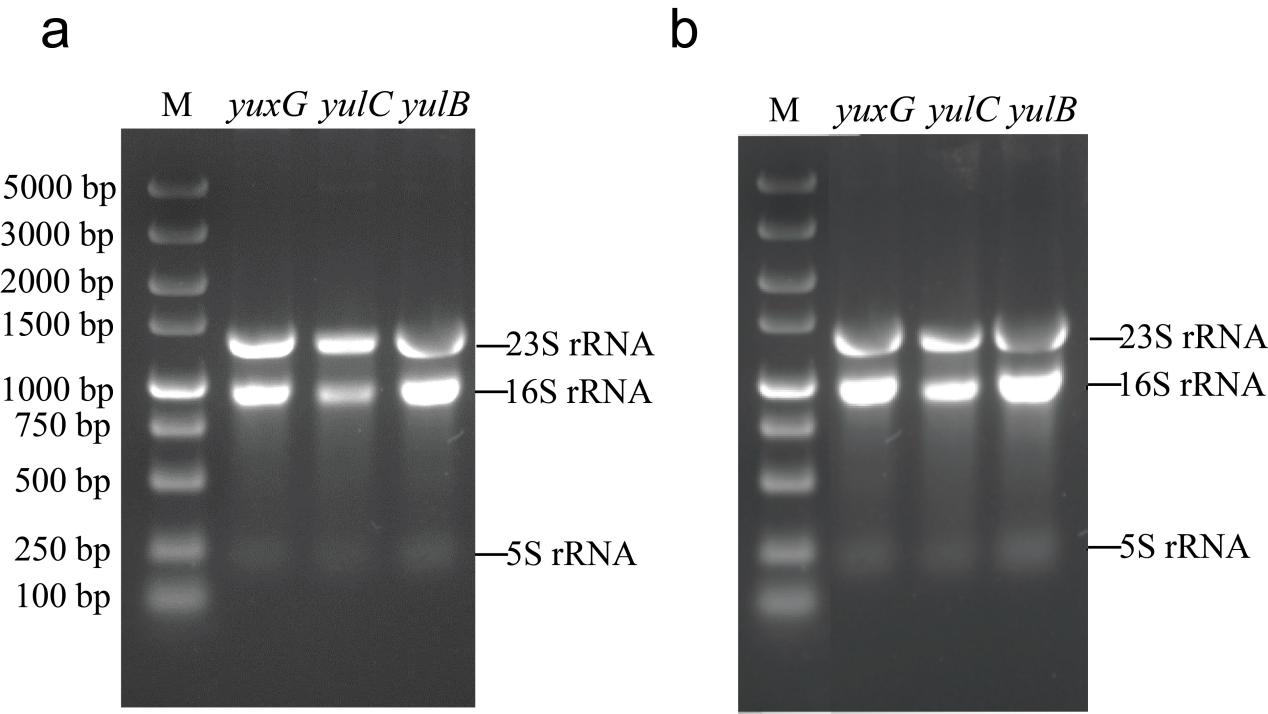


**Supplementary Figure 2.** Total RNA extracted from *B. licheniformis* cultured in LBG (a) and LBR (b) media.


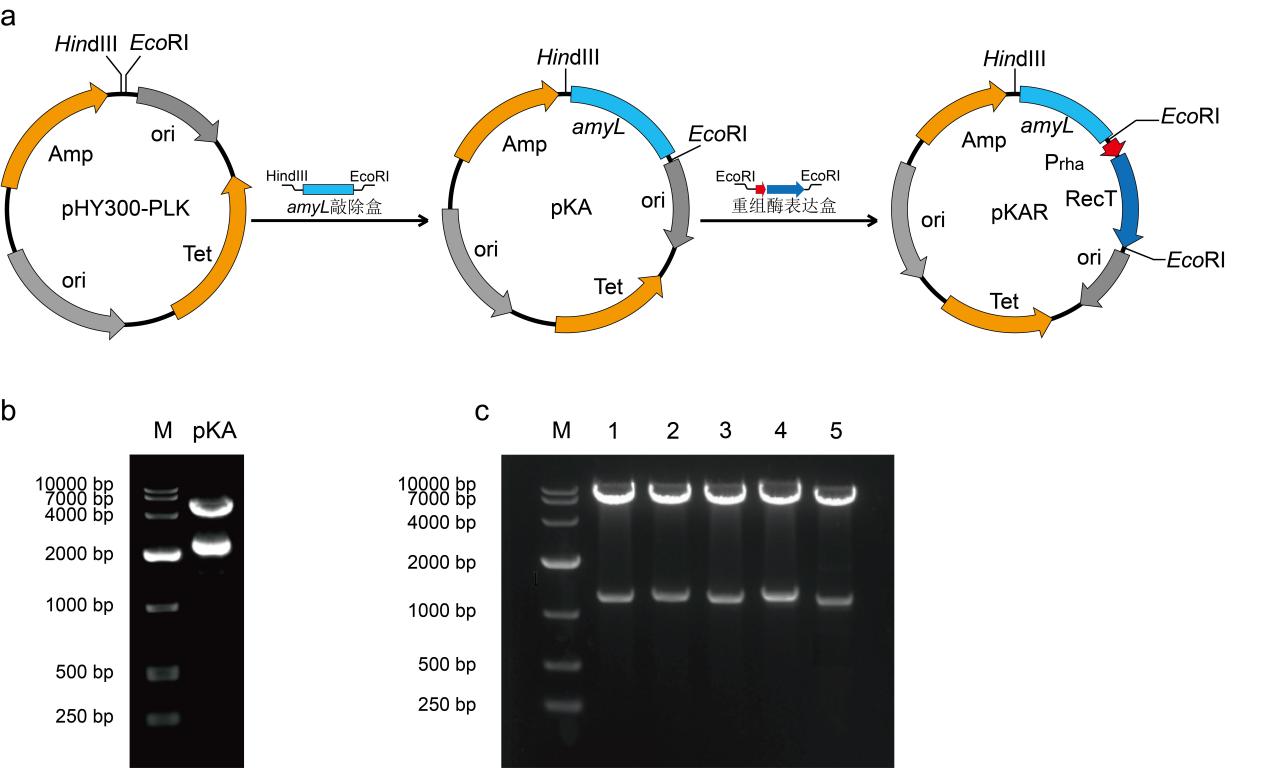


**Supplementary Figure 3.** Construction scheme of genome editing plasmid pKAR (a). Gel analysis of the plasmid pKA digested by *Eco*RI and *Hind*III (b). Gel analysis of the plasmid pKAR1-5 digested by *Eco*RI (c).
